# Preoperative anxiety and health literacy in patients applying to the anesthesia outpatient clinic

**DOI:** 10.1093/eurpub/ckac130.185

**Published:** 2022-10-25

**Authors:** M Mutlu, A Mutlu, D Oktar, B Yelken, D Arslantas, A Unsal

**Affiliations:** 1 Anesthesiology and Reanimation, Osmangazi University Faculty of Medicine, Eskisehir, Turkey; 2 Public Health, Osmangazi University Faculty of Medicine, Eskisehir, Turkey

## Abstract

**Background:**

Evaluation of preoperative anxiety and health literacy is important for healthcare professionals to understand the needs and expectations of patients and provide them with the necessary support. The study aimed to evaluate the preoperative anxiety levels of patients who applied to the anesthesia outpatient clinic and its relationship with health literacy.

**Methods:**

A cross-sectional study was conducted on patients who applied to the anesthesia outpatient clinic of Eskişehir Osmangazi University Medical Faculty Hospital in March 2022. The questionnaire form, which was prepared by making use of the literature, was filled in by face-to-face interview method after obtaining the participants’ verbal consent. The 6 item Amsterdam Preoperative Anxiety and Information Scale (APAIS) in which the scores that can be taken range from 6 to 30 and higher score means higher anxiety was used to assess the level of anxiety, and the 16 item European Health Literacy Scale Short Form was used to assess health literacy. Descriptive statistics, Chi-Square test and Logistic Regression analysis were used to analyze the data.

**Results:**

In the study group, 197 (50.3%) were female. Their ages ranged from 18 to 86, with a mean of 45.7 ± 17.2 years. The median (min-max) APAIS score was 15 (6-30). There was a moderate negative correlation between the scores obtained from the APAIS and the European Health Literacy Scale Short Form (r= -0.50, p < 0.01). According to the logistic regression analysis, preoperative anxiety was found to be 1.53 (%95 CI; 1.01-2.30) times higher in women, 3.49 (%95 CI; 1.23-9.94) times higher in those with low family income, and 1.61 (%95 CI; 1.07-2.42) times higher in those with type A personality.

**Conclusions:**

More than half of the patients had preoperative anxiety. The level of preoperative anxiety decreased as the health literacy level increased.

**Key messages:**

- Necessary support should be given for the preoperative anxiety of the patients.
- Public health policies should be developed in order to increase health literacy.

